# Impact and Outcomes of Pretreatment Total Serum Testosterone on Localized Prostate Cancer Patients

**DOI:** 10.1155/2020/8357452

**Published:** 2020-01-20

**Authors:** Brittni M. Usera, Polly Creveling, Jonathan D. Tward

**Affiliations:** ^1^Department of Radiation Oncology, University of California at Davis, Davis, CA, USA; ^2^Cancer Control and Population Sciences, Huntsman Cancer Institute, University of Utah, Salt Lake City, UT, USA; ^3^Department of Radiation Oncology, Huntsman Cancer Institute, University of Utah, Salt Lake City, UT, USA

## Abstract

**Purpose:**

To investigate how pretreatment testosterone levels correlate with progression-free survival, metastasis-free survival, and overall survival in a propensity-adjusted localized prostate cancer population.

**Methods:**

Men diagnosed with clinical NCCN-risk stratified very-low, low, intermediate, high, and/or very-high risk prostate cancer who had a baseline total serum testosterone level≥100 ng/dl measured within the 100 days preceding the first definitive therapy were identified from our prospectively gathered institutional database. Cohorts below (100–239 ng/dl), within (240–593 ng/dl), or above (594 + ng/dl) one standard deviation from the mean testosterone level (416 ng/dl) were used for comparison. Progression-free, metastasis-free, and overall survival were evaluated. A separate cohort of men not receiving ADT was used to evaluate testosterone recovery after various treatment modalities (surgery, external beam radiation, brachytherapy, or combined EBRT + Brachy).

**Results:**

There was no statistically significant difference between the low, average, and high testosterone cohorts for PFS, MFS, or OS. In men not using ADT, there were no statistically significant changes in testosterone levels 1 year after therapy, regardless of therapy type.

**Conclusion:**

In men with serum testosterone levels >=100 ng/dl at diagnosis, baseline testosterone does not impact PFS, MFS, or OS. Recovery of testosterone back to baseline is expected for men undergoing either surgery, external beam or brachytherapy, or combined modality radiation when not using ADT.

## 1. Introduction

In the 1940s, Huggins and Hodges discovered that ADT led to the regression of metastatic prostate cancer [1]. This observation, and others from in vitro work, led to the androgen hypothesis of prostate cancer pathogenesis, which theorizes that high androgen concentration increases prostate cancer risk and low testosterone was protective [2]. Over the past few decades, numerous researchers have investigated the correlation of pretreatment serum testosterone levels with cancer aggressiveness at diagnosis and clinical outcomes. The published data in this regard has been conflicting, and a recently published review article on the matter has concluded that “much of the controversy appears to be based on conflicting study designs, definitions and methodologies” [3].

The majority of studies evaluating the relationship between pretreatment testosterone and staging or oncologic outcomes have been performed in men who received a radical prostatectomy. Fewer studies have been performed in men who have received radiation therapy [4–11]. None of the published studies to date have evaluated the correlation between pretreatment serum testosterone and metastasis-free survival, or how combined modality radiations like EBRT and brachytherapy correlate with outcomes stratified by pretreatment testosterone levels.

The aim of this study was to investigate how pretreatment testosterone levels correlate with biochemical failure-free survival, metastasis-free survival, and overall survival in a propensity-adjusted population that accounts for the NCCN-risk group [12] (version 2.2019), type of radiation or surgical therapy delivered, and use of neoadjuvant, concurrent, or adjuvant ADT. An additional aim of the study was to characterize how the various treatments studied affected posttreatment testosterone levels in men not receiving ADT.

## 2. Methods

### 2.1. Patient Cohort Selection

The Huntsman Cancer Institute at the University of Utah has a prostate cancer outcome database that was abstracted retrospectively of patients presented to the University of Utah prior to 2012 and prospectively since that time. Data included in the database are abstracted by professional GU oncology tumor registrars and are populated by direct import of lab values from the electronic medical record and by computerized natural language processing algorithms used to automatically abstract relevant TNM, Gleason, PSA, and Testosterone values from free-text provider notes with 99% or greater data fidelity. All data used in this project were further audited and validated by an attending physician specialized in the treatment of GU malignancies. For additional details about the data abstraction, auditing, and validation methods, see Supplementary Materials.

Study inclusion criteria included men diagnosed with clinical NCCN-risk stratified very-low, low, intermediate, high, and/or very-high risk prostate cancer who had a baseline total serum testosterone level ≥100 ng/dl measured within the 100 days preceding the first definitive therapy. All testosterone measurements were performed using the quantitative electro-chemiluminescent immunoassay. Men were excluded from the analysis if they could not be NCCN-risk stratified, or if the details of their definitive radiation or surgical therapies, and ADT use prior to or after therapy were unknown. The mean pretreatment total serum testosterone value of the study cohort was 416 ng/dl.

Low (100–239), normal (240–593), and high (594+) baseline testosterone cohorts were defined as 1 standard deviation below, within, or above the study population mean.

### 2.2. Definitions of Failure

All failure definitions were timed from the start date of either surgery or radiation therapy. Biochemical failure was defined for radiation therapy patients as the PSA nadir +2 ng/ml and for surgical patients as a postoperative PSA ≥0.2 ng/ml, or the receipt of adjuvant or salvage therapy if that PSA threshold had not yet been reached. Progression-free survival (PFS) included biochemical failure, local recurrence, or metastasis. Metastasis was defined as any nonregional nodal adenopathy, bone or visceral metastases occurring 2 months or more after completion of definitive therapy. Death from any cause was used for overall survival calculations.

### 2.3. Statistical Analyses

#### 2.3.1. Propensity Score Adjustment

Due to the inherent imbalance of known prognostic factors within the low-, normal-, and high-testosterone cohorts, propensity-adjustment and/or Cox regression were used in the outcome analyses. A generalized boosted model was used to compute propensity weights for individuals using the dependent variables of the NCCN-risk group, type of curative treatment attempted, age at treatment, and use of neoadjuvant, concurrent, or adjuvant ADT to surgery or radiotherapy using the Rand Corporation Toolkit for Weighting and Analysis of Nonequivalent Groups (TWANG) package of Stata (version 15, Stata LLC) statistical software. [13] Standardized mean differences were used to evaluate the balance of prognostic covariates, and values ≤0.2 were considered well balanced, and those ≤0.1 were considered extremely well balanced.  Table 1 reveals the unweighted and propensity-weighted balance of patients in each pretreatment testosterone cohort stratified by the NCCN-risk group, type of definitive therapy used, and use of neoadjuvant, concurrent, and/or adjuvant ADT use.

#### 2.3.2. Survival Estimates and Analyses

Propensity-adjusted Kaplan–Meier curves were used to graphically evaluate and estimate survival. Because overall survival is known to be influenced by comorbidity and coronary artery disease, a doubly robust model incorporating propensity weighting and Cox regression was used to estimate hazard ratios and statistical significance for overall survival in the propensity-adjusted regressions [14].

Median follow-up for survival endpoints was calculated using the reverse Kaplan–Meier method [15]. The median follow-up time for PFS, MFS, and OS was 4.2 years.

#### 2.3.3. Testosterone Trends over Time Analyses

A subgroup of individuals who never received ADT before or after definitive therapy was identified and used to evaluate the effect of therapy type on testosterone levels after treatment. Effects were evaluated using linear regression of the means and a random-effects regression model accounting for how individuals progressed over time, the treatment received, and age at diagnosis and NCCN-risk group.

## 3. Results

### 3.1. Comparisons of Clinical Characteristics of Treatment Cohorts

The clinical demographic features of the 258 patients included in the analysis stratified by pretreatment testosterone cohort are shown in Table 1. After propensity weighting, there was still a small amount of imbalance (standardized difference >0.2) noted for the surgery groups between the low testosterone cohort and the average and high testosterone cohorts; however, this imbalance did not reach statistical significance on the chi-squared analysis (Table 1).

### 3.2. Estimates of PFS, MFS, and OS

**Figure 1:**
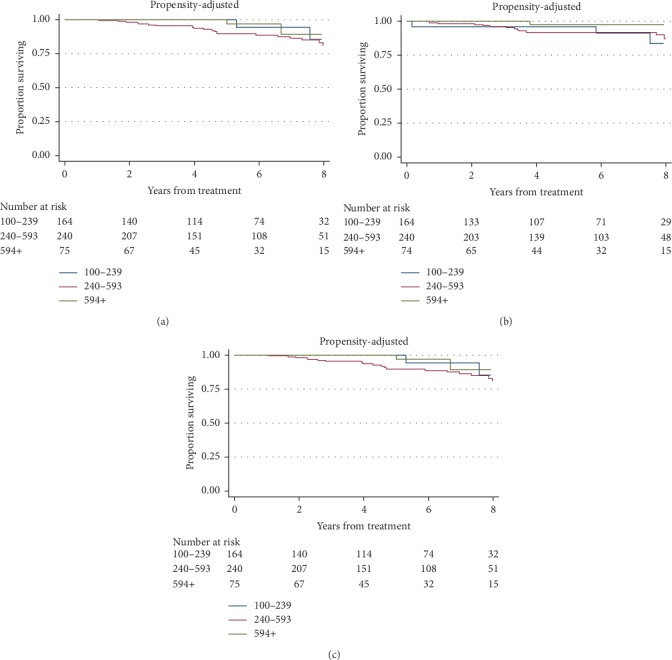
Propensity-adjusted Kaplan–Meier curves for progression fee (a), metastasis-free (b), and overall survival (c). The numbers at risk for each cohort represent the propensity-weighted population size. The actual population size for the 100–239, 240–593, and 594 +  cohorts is actually 39, 176, and 43 persons at baseline.

During the study period, 28 deaths, 19 metastatic events, and 50 progression events were observed. The propensity-adjusted 5-year PFS for the low-, average-, and high-testosterone cohorts was 90.6%, 80.0%, and 81.0%, respectively. The 5-year MFS for the low-, average-, and high-testosterone cohorts was 93.9%, 100%, and 98.3%, respectively. The 5-year OS for the low-, average-, and high-testosterone cohorts was 100%, 88.7%, and 92.3%, respectively. The propensity-adjusted Kaplan–Meier survival curves are shown in Figure 1 (unadjusted curves shown in supplementary Figure 1).

### 3.3. Propensity-Adjusted and Unadjusted Multivariable Hazard Ratios for PFS, MFS, and OS

Hazard ratios for survival outcomes were analyzed independently using propensity adjustment and multivariable Cox regression. There was no statistically significant difference between the low-, average-, and high-testosterone cohorts for PFS, MFS, or OS (Table 2). The presence of ISUP grade group 4 or 5 (Gleason 9 or 10) biopsy pathology was associated with significantly worse MFS (hazard ratio 4.9, 95% CI 1.1 to 22.2, *p*=0.04) compared to patients with a grade group of 1 or 2 (Gleason 3 + 3 or 3 + 4). Patients with clinically staged extracapsular extension (T3a) or seminal vesicle invasion (T3b) were at a higher risk of progression (HR = 4.9, 95% CI 1.2 to 19.6, *p*=0.03) and had a trend toward increased death (HR = 7.3, 95% CI 0.9 to 56.6, *p*=0.06) than those whose cancer was discovered due to elevated PSA (clinical T1c) alone.

### 3.4. Effect of Treatment Modality on Posttherapy Testosterone Levels

**Figure 2:**
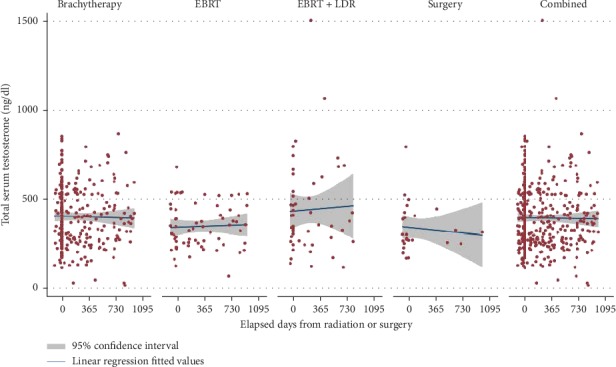
Testosterone trends by treatment cohort over time.

**Figure 3:**
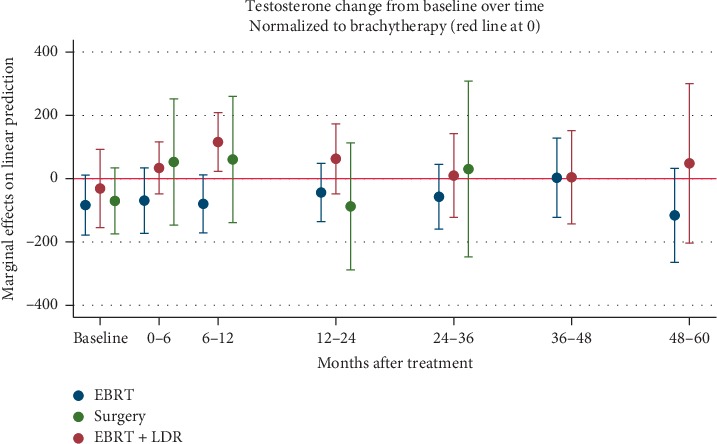
Marginal effects modelling of mean changes of testosterone over time by treatment cohort, normalized to 0 for the baseline mean of the brachytherapy cohort. Error bars represent standard deviations from the mean.

Linear regression analysis did not reveal any statistically significant long-term changes in testosterone levels before or after therapy, regardless of therapy type (Table 3 and Figure 2). Likewise, comparisons of one therapy type to another using an average marginal random-effects model adjusting for patient age and pretreatment NCCN-risk group did not reveal any significant differences for pre-vs posttherapy testosterone levels after year 1 (Figure 3).

## 4. Discussion

Numerous studies have been performed over the past few decades with the aim of characterizing how pretreatment testosterone affects staging, prognosis, and outcomes. Many are contradictory. An extensive contemporary review by Klap et al. sheds light on the limitations of the existing literature, which variously reveals studies limited by small sample size, retrospective nature, insufficient follow-up, measurements of testosterone not adhering to professional society guidelines, and poor quality statistical design [3]. In our study, we attempted to minimize some of the limitations of these other studies by using data from a prospectively gathered institutional database of all prostate cancer patients (minimizing selection bias), and by using a rigorous statistical approach that attempted to control for the confounding of other known prognostic indicators and various treatment modalities. To the best of our knowledge, this is the first study in a propensity-adjusted population to evaluate how pretreatment testosterone levels correlate with progression-free survival, metastasis-free survival, and overall survival that accounts for NCCN-risk group, type of radiation or surgical therapy delivered, and use of neoadjuvant, concurrent, or adjuvant ADT.

In our study, over 90% of patients were treated with various definitive radiation mono or combined modality therapies as opposed to surgery. This is largely due to the routine clinical practice of the radiation oncology group at our institution measuring baseline serum testosterones along with pretreatment PSA values. The primary clinical rationale for this institutional standard was to assess if the baseline PSA value could be influenced by undiagnosed hypogonadism. In a prospective study of 2162 males over the age of 45, 38.7% had hypogonadism (defined as total testosterone <300 ng/dl) [16]. Approximately, 10% had levels below 200 ng/dl and around 4% had levels <100 ng/dl. The risk of hypogonadism increase increases with age, obesity, diabetes, and persons with a prostatic disease or disorder [16]. Given that the average age of a newly diagnosed prostate cancer patient in our study was approximately 64 years, one would anticipate that about 1 in 20 men would present with severe hypogonadism. The use of androgen deprivation therapy with unfavorable intermediate and high-risk prostate cancer has been correlated on prospectively randomized trials with improved PFS, MFS, and OS over radiation therapy alone [17–22]. However, its use is also correlated with significant toxicity and degradation of quality of life [23]. The additional cost to the health-care system by using ADT with radiation is approximately $17,000 in the first year [24]. Therefore, identifying men who would otherwise require ADT but are incidentally severely hypogonadal at baseline could spare them from receiving additional ADT, which would be futile and costly. Although severe hypogonadism is an uncommon presentation, men in this category could be considered to have castrate-resistant disease, in which case there would be discussion among a multidisciplinary group about using systemic therapies alone, using second-generation antiandrogens with localized therapy or possibly chemotherapy [25–27]. Testosterone was also routinely measured at follow-up to assess if a report of fatigue after radiation therapy could correlate with changes from baseline. Fatigue is one of the most common side effects reported in cancer patients receiving radiation therapy for prostate cancer, which can persist for many months [28].

We found no significant correlation between pretreatment testosterone level and ISUP grade group, or on NCCN clinical risk stratification. Although other institutions have (paradoxically) correlated adverse stages and grades both with high- and low-serum total testosterone concentrations [3], our result is consistent with the findings of a thorough meta-analysis of 18 prospective studies that included 3886 men with prostate cancer, which revealed no correlation of serum testosterone concentrations with either stage or grade of disease [29].

Although numerous studies have evaluated the relationship between baseline serum testosterone concentration and the risk of biochemical failure, we could not identify other studies that evaluated MFS or OS in a primarily irradiated population. Additionally, most studies with biochemical failure have been done in those receiving radical prostatectomy [2, 3, 29], with far fewer studies in those receiving radiation therapy [7, 9]. We found that serum testosterone concentrations lower or higher than 1 standard deviation from the mean had no significant impact on these outcomes.

Prospective, placebo controlled studies have demonstrated that men on testosterone replacement therapy (TRT) prior to diagnosis are not at elevated risk of developing prostate cancer [30]. Although we cannot be certain that prior testosterone replacement therapy does not have an effect on oncologic outcomes, a small series of 13 men who were using TRT while simultaneously on active surveillance (12 with Gleason 6 and 1 with Gleason 7) suggests that continued use of TRT was not associated with cancer progression more so than might be expected for a typical man on active surveillance [31]. These previous findings, coupled with our observation that pretreatment testosterone levels do not seem to impact important oncologic outcomes after therapy, may suggest that prior TRT is unlikely to impact the probability of achieving cancer control after therapy. This hypothesis however, would have to be examined in a separate work, as the current study did not analyze the impact of prior TRT on outcomes.

One of the limitations of this study is that serum testosterone concentration was not uniformly measured in the morning as is suggested by consensus guidelines [32]. Given that there is significant known diurnal variation of testosterone levels, patients near our cohort testosterone value thresholds may find themselves moving between our cohorts throughout the day with a circadian rhythm. Diver et al. categorized this rhythm, and their work implies that levels typically fluctuate about ±25% around the mean daily testosterone level within any given day [33]. Nevertheless, we would anticipate that if there was a clinically significant effect, one might see it between the lowest and highest testosterone cohorts in our study, where there would be no overlap in these diurnal variations. Since these cohorts were not significantly different from one another for PFS, MFS, or OS, our conclusions remain the same.

In conclusion, the heterogeneity of baseline serum testosterone values above the castrate range at diagnosis do not seem to alter the risk of progression, metastasis, or death following prostate cancer-directed curative intent interventions when accounting for more relevant clinical parameters like ADT use, NCCN-risk group, ISUP grade group, age, T-stage, and medical comorbidities. This work provides additional assurances that transient declines in testosterone levels following various forms of radiation therapy, including combined modality EBRT and brachytherapy will resolve within 1 year if not using androgen deprivation therapies.

## Figures and Tables

**Table 1:** Clinical demographic features of the study population.

	All patients	Unweighted baseline cohort									Propensity-weighted cohort					
		Testosterone cohort			Absolute standardized difference			*P* value			Absolute standardized difference			*P* value		
*Cohort*		**1**	**2**	**3**	**1vs2**	**1vs3**	**2vs3**	**1vs2**	**1vs3**	**2vs3**	**1vs2**	**1vs3**	**2vs3**	**1vs2**	**1vs3**	**2vs3**

	**Total**	**100–239**	**240–593**	**594+**												

*# of patients*	258	39	176	43												
*Age (yrs)*		63.9	65.4	64.5	0.20	0.08	0.12	0.21	0.69	0.48	0.11	0.03	0.08	0.56	0.89	0.64
	**%**	**%**	**%**	**%**												
*ADT used with therapy*																
ADT used	19	10.3	18.8	27.9	0.21	0.44	0.23	0.21	0.05	0.19	0.16	0.16	0.00	0.47	0.07	0.98
No ADT	81	89.7	81.3	72.1	0.21	0.44	0.23				0.16	0.16	0.00			

*NCCN-risk group*																
High/VH risk	20.9	23.1	21	18.6	0.05	0.11	0.06	0.29	0.89	0.27	0.10	0.00	0.10	0.85	0.97	0.72
Intermediate risk	50.4	41	54	44.2	0.26	0.06	0.20				0.01	0.05	0.06			
Low risk	28.7	35.9	25	37.2	0.24	0.03	0.27				0.09	0.06	0.15			

*Therapy*																
Brachytherapy	57.8	59	56.8	60.5	0.04	0.03	0.07	0.62	0.14	0.40	0.15	0.1	0.05	0.60	0.70	0.95
EBRT	16.3	23.1	17	7	0.17	0.45	0.28				0.02	0.12	0.11			
EBRT + LDR	14	7.7	14.2	18.6	0.18	0.31	0.12				0.03	0.04	0.01			
Surgery	12	10.3	11.9	14	0.05	0.11	0.06				0.21	0.24	0.03			

*ISUP grade group*																
Group 1,2		76.9	68.8	65.1	0.18	0.25	0.08	0.19	0.26	0.82						
Group 3		5.1	16.5	16.3	0.31	0.30	0.01									
Group 4,5		17.9	14.8	18.6	0.09	0.02	0.11									

*Clinical T-stage*																
T1c	20.9	51.3	63.6	55.8	0.26	0.09	0.16	0.25	0.92	0.45						
T2	50.4	41	33	37.2	0.17	0.08	0.09									
T3a/b	28.7	7.7	3.4	7	0.22	0.04	0.18									

*Body mass index*																
25–29.9	18.6	20.5	17.6	20.9	0.08	0.01	0.09	0.9119	0.53	0.338						
30+	15.9	15.4	18.2	7	0.08	0.23	0.31									
<25	7.4	7.7	6.8	9.3	0.03	0.06	0.10									
Unknown	58.1	56.4	57.4	62.8	0.02	0.04	0.05									

*Smoking history*																
Current	7	7.7	5.1	14	0.10	0.25	0.35	0.54	0.57	0.24						
Former	24.8	33.3	23.9	20.9	0.22	0.29	0.07									
Never	50.4	43.6	52.3	48.8	0.17	0.11	0.07									
Unknown	17.8	15.4	18.8	16.3	0.09	0.02	0.06									

*Pre-treatment PSA*																
10–20	32.2	41	31.3	27.9	0.21	0.29	0.07	0.43	0.46	0.79						
<10	59.7	53.8	59.7	65.1	0.12	0.23	0.11									
>20	8.1	5.1	9.1	7	0.14	0.07	0.08									

*P* values for the demographics are calculated based on the chi-squared test. Clinical T-stage derived from the American Joint Committee on Cancer (AJCC) 8^th^ edition. Comparison of standardized difference for the unweighted and propensity weight populations used in the analyses are shown. EBRT = external beam radiation therapy, VH=very high, GS = Gleason score, ADT = Androgen deprivation therapy, ISUP = International Society of Urological Pathologists.

**Table 2:** Propensity-adjusted and multivariable adjusted regression analyses for survival endpoints.

Propensity-adjusted Cox regression									
	PFS			MFS			OS		
	Hazard ratio	95% CI	*P* value	Hazard ratio	95% CI	*P* value	Hazard ratio	95% CI	*P* value

*Testosterone cohort*									
100–239	0.71	0.28 to 1.80	0.47	1.31	0.34 to 5.03	0.70	0.99	0.35 to 2.83	0.98
240–593	Reference			Reference			Reference		
594+	0.78	0.35 to 1.74	0.55	0.62	0.14 to 2.72	0.53	0.75	0.26 to 2.11	0.58

*NCI comorbidity score*							3.06	0.44 to 21.23	0.26

*Coronary artery disease*									
No							Reference		
Yes							0.86	0.19 to 3.84	0.84

Multivariable Cox regression									
	PFS			MFS			OS		
	Hazard ratio	95% CI	*P* value	Hazard ratio	95% CI	*P* value	Hazard ratio	95% CI	*P* value

*Testosterone cohort*									
100–239	0.74	0.29 to 1.88	0.53	1.34	0.32 to 5.60	0.69	0.86	0.27 to 2.74	0.81
240–593	Reference			Reference			Reference		
594+	0.48	0.19 to 1.19	0.11	0.41	0.08 to 2.22	0.30	0.53	0.13 to 2.19	0.38

*Age (yrs)*	1.0	0.96 to 1.06	0.85	1.02	0.95 to 1.10	0.57	1.06	0.99 to 1.13	0.08

*ISUP grade group*									
Group 1,2	Reference			Reference			Reference		
Group 3	1.4	0.54 to 3.59	0.49	1.62	0.28 to 9.44	0.59	1.06	0.29 to 3.83	0.93
Group 4,5	1.7	0.65 to 4.44	0.28	4.85^*∗*^	1.06 to 22.24	0.04	1.77	0.43 to 7.40	0.43

*Log (psa)*	1.5	0.91 to 2.48	0.11	1.47	0.65 to 3.33	0.35	1.31	0.64 to 2.68	0.46

*Clinical T-stage*									
T1c	Reference			Reference			Reference		
T2	0.72	0.36 to 1.44	0.36	0.35	0.09 to 1.30	0.12	1.03	0.42 to 2.51	0.95
T3a/b	4.85^*∗*^	1.20 to 19.60	0.03	1.39	0.25 to 7.72	0.71	7.28	0.94 to 56.64	0.06

*NCI comorbidity score*							2.65	0.43 to 16.27	0.29

*Coronary artery disease*									
No							Reference		
Yes							0.91	0.17 to 4.78	0.92

Clinical T-stage derived from the American Joint Committee on Cancer (AJCC) 8^th^ edition. PFS = progression-free survival, MFS = metastasis-free survival, OS = overall survival, ISUP = International Society of Urological Pathologists, NCI = National Cancer Institute.

**Table 3:** Predicted change over time relative to baseline testosterone level by treatment modality. The change was determined by linear regression modelling.

Therapy	Predicted change in testosterone level/year from baseline	95% confidence interval	*P* value	*R*-squared	Statistical interpretation
Brachytherapy	0.17	−12.9 to 13.2	0.979	<0.001	No significant change
EBRT	4.8	−10.3 to 19.8	0.529	0.005	No significant change
EBRT + LDR	−13.2	−0.6 to 24.1	0.482	0.008	No significant change
Surgery	−20.5	−88.7 to 47.6	0.54	0.016	No significant change
All treatments combined	−0.0	−0.03 to 0.02	0.780	<0.001	No significant change

## Data Availability

The clinical data used to support the findings of this study are restricted by the University of Utah Institutional Review Board in order to protect patient privacy. Data are available from the corresponding author for researchers who meet the criteria for access to confidential data.

## Supplementary Materials

Supplementary Materials Supplementary Figure 1: Kaplan–Meier curves for progression, metastasis, and death in the unadjusted population. Supplementary methods: detailed methods regarding the automation, natural language processing, and auditing of the source data.
