# Supplementary material for: Impact and Outcomes of Pretreatment Total Serum Testosterone on Localized Prostate Cancer Patients
Source: Prostate Cancer. 2020 Jan 20;2020:8357452. doi: 10.1155/2020/8357452 (PMC7201500; doi:10.1155/2020/8357452)
Supplement: Supplementary Materials — Supplementary Figure 1: Kaplan–Meier curves for progression, metastasis, and death in the unadjusted population. Supplementary methods: detailed methods regarding the automation, natural language processing, and auditing of the source data. [file 8357452.f1.pdf]

Supplementary Figure 1

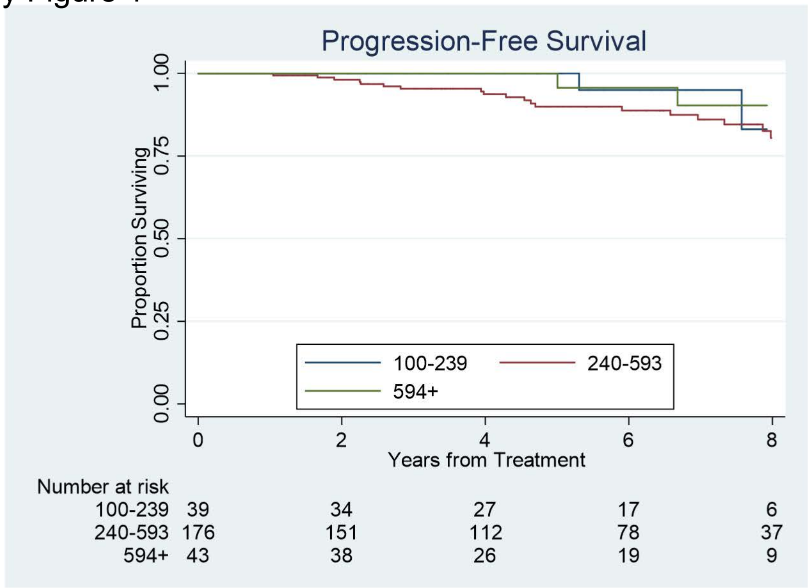

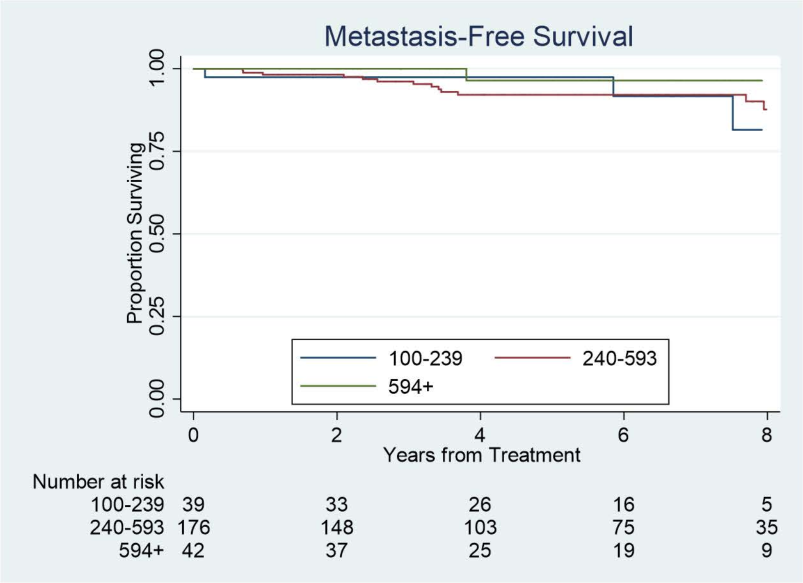

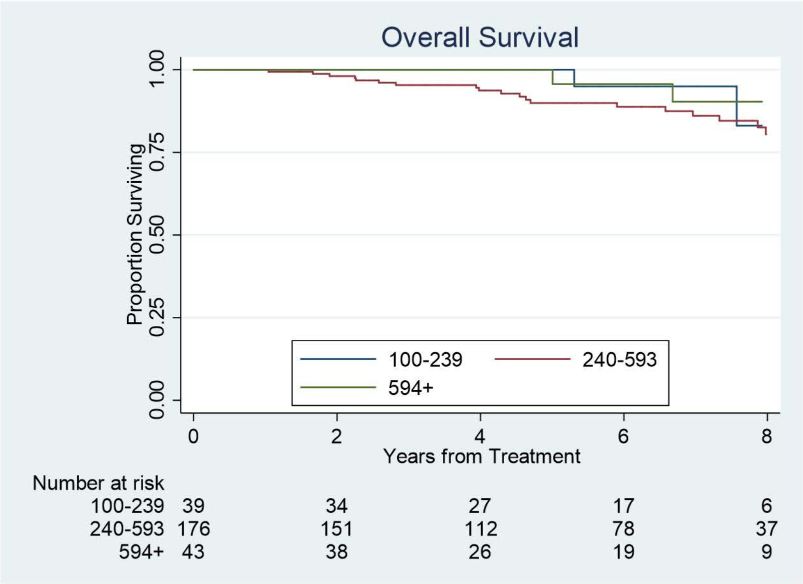

Supplementary Methods Section

Additional Description of Data Acquisition Methods

Baseline data fidelity, categorization, and abstraction

In 2012, the University of Utah formalized its processes to both retrospectively and prospectively gather comprehensive outcomes data and baseline characteristics on all subjects diagnosed with prostate cancer seen at the University over the past 20 years. The institutional review board has approved this research. Subjects definitively treated for NCCN high or very high-risk prostate cancer treated after Jan 1, 2000 were included in the study. During this timeframe, the University Healthcare System has utilized electronic medical records systems.

Case Ascertainment:

A robust institutional cancer clinical research database (CCR) with over 12,000 individuals diagnosed with prostate cancer was initially created by merging records within the University healthcare system by utilizing all the following methods:

1. Individual investigators outcomes databases from previously approved prostate cancer research projects were unified into the CCR database.
2. The University healthcare system has collected ICD codes for all medical diagnoses from it's electronic medical record systems from 2000 onward. All subjects with an ICD code of 185 (prostate cancer) were retrospectively identified and merged into the database. Beginning in 2015, all new ICD codes of 185 have been automatically added daily.
3. The electronic data warehouse (EDW) is a centralized repository of all free-text clinical notes, imaging and pathology reports, CPT encoding, and discrete data such as laboratory values obtained University Healthcare system-wide sourced from the various electronic medical records systems formerly and currently used by the University Hospitals and clinics. Natural language processing algorithms are routinely run to identify subjects with prostate cancer by looking for terms such as "Gleason", "PSA", or "Prostate Cancer"

Data abstraction and fidelity

Several methods were utilized to ensure high data quality and completeness for this study:

1. The University Healthcare system has been required by the state of Utah administrative code to collect and manage cancer incidence and mortality data since 1966. The University cancer registry has professional data abstractors who collect clinical TNM staging, and details of therapy. Since 1973 Utah has contributed to the NCI – SEER program and these same data elements collected by that program are available to the University under IRB approval. All data from the University cancer registry is periodically audited by specialist physicians to ensure accurate encoding.
2. Discrete data elements such as all PSA and Testosterone lab values are automatically imported into the CCR database daily from the clinical electronic medical records.

3. Natural language processing algorithms have been developed to automatically abstract clinical and pathological TNM staging, PSA and testosterone values, and biopsy and surgical pathologic Gleason scores. All NLP methods have been validated by expert physician reviewers to ensure >99% accuracy with correct date-context before release into the CCR database
4. The Team employs 2 fulltime professional data abstractors to retrospectively and prospectively collect and validate data elements not routinely collected by the cancer registry and items not found by natural language processing. These include but are not limited to comorbidity data, laboratory values, details of surgical, radiation and systemic therapies, details of imaging studies and molecular testing, and patterns of treatment failure. The Team employees also validate the accuracy of all tumor registry abstractors, and in the case of data discrepancies between medical notes, will review the source material to determine the most accurate representation of the data.
5. NCCN clinical risk group for all subjects in the database is automatically calculated nightly and assigned to the subject record. Subjects without a calculated NCCN risk group are given to the professional data abstractors to resolve.
6. Subjects with NCCN High or Very High Risk, who were definitively treated by University expert urologists or radiation oncologists were included in the study. Subjects initially treated by outside physicians were excluded from the study.
7. The corresponding author personally audited every subject record to ensure accurate TNM staging, Gleason score, treatment details, medical comorbidities, failure patterns and NCCN risk assignment. As such, each treatment record was reviewed and validated by a professional tumor registrar, a professional prostate cancer specific data-abstractor from the Team, and an attending physician expert specializing in the management of prostate cancer.
